# Right-sided rhabdoid colorectal tumors might be related to the Serrated Pathway

**DOI:** 10.1186/1746-1596-8-31

**Published:** 2013-02-20

**Authors:** Massimo Pancione, Andrea Remo, Lina Sabatino, Caterina Zanella, Carolina Votino, Alessandra Fucci, Arturo Di Blasi, Giovanni Lepore, Bruno Daniele, Francesca Fenizia, Enrico Molinari, Nicola Normanno, Erminia Manfrin, Roberto Vendraminelli, Vittorio Colantuoni

**Affiliations:** 1 Department of Biological, Geological and Environmental Sciences, University of Sannio, Via Port’Arsa, Benevento, 11 82100, Italy; 2 Department of Pathology “Mater Salutis” Hospital, ULSS21, Legnago, Verona, Italy; 3 Departments of Oncology and Pathology, Azienda Ospedaliera “G. Rummo”, Benevento, 82100, Italy; 4 Center for Oncology Research, Pharmacogenomic Laboratory, Mercogliano, Avellino, 83013, Italy; 5 Department of Surgery “Mater Salutis” Hospital, ULSS21, Legnago, Verona, Italy; 6 Department of Pathology “G.B. Rossi” Hospital, University of Verona, Verona, Italy

**Keywords:** Rhabdoid Colorectal Tumor, RCT, CpG island methylator phenotype, CIMP, Serrated pathway

## Abstract

**Background:**

Rhabdoid colorectal tumor (RCT) is a rare, highly aggressive neoplasm recurrent in elderly patients, commonly at the caecum. The molecular mechanisms underlying RCT pathogenesis remain poorly elucidated. The differential diagnosis is with the malignant rhabdoid tumors of infancy characterized by genetic inactivation of *SMARCB1* (INI1) or deletions of chromosome 22q12 locus.

**Materials and methods:**

To shed light on RCT pathogenesis, we investigated genetic and epigenetic alterations in two cases of pure and composite RCT and compared them with the profiles of matched adenomas and normal mucosa. Immunohistochemical analysis, FISH, methylation specific PCR and DNA sequencing analysis were performed on paraffin-embedded tissues.

**Results:**

Loss of epithelial markers, (CK20, CDX2 and E-cadherin) and intense vimentin expression was observed in RCTs but neither in the normal mucosa or adenomas. *INI1* expression was detected in normal mucosa, adenomas and retained in pure RCT, while it was undetected in composite RCT. Rearrangement of the 22q12 locus was found only in pure RCT. The APC/β-catenin pathway was not altered, while MLH1 immunostaining was negative in RCTs and positive in adenomas and normal mucosa. These expression profiles were associated with V600E *BRAF* mutation, a progressive accumulation of promoter methylation at specific CIMP loci and additional genes from the normal mucosa to tubular adenoma and RCT.

**Conclusions:**

Right-sided RCT could be characterized by epigenetic events and molecular features likely similar to those occurring in the serrated pathway and associated with epithelial-mesenchymal transition. These extremely rare tumors may benefit from the use of new biological molecules specific for colorectal carcinoma.

**Virtual slides:**

The virtual slide(s) for this article can be found here: http://www.diagnosticpathology.diagnomx.eu/vs/1641385210804556

## Background

Rhabdoid colorectal tumor (RCT) is a rare lesion mainly localized to the proximal colon in patients with a mean age at diagnosis around 70 years. Only 7 cases of RCT have been reported in the literature to the best of our knowledge [1-3]. This tumor shows an aggressive behaviour and fatal outcome displaying an overall survival shorter than 12 months [1-3]. The diagnostic hallmark of this neoplasm is the presence of rhabdoid cells characterized by an eccentrically located and large nucleus, prominent nucleoli and cytosolic aggregates of intermediate filaments [1-3]. The amount and distribution of the rhabdoid component in neoplasms is highly variable ranging from “composite,” in which the rhabdoid elements are associated with adenocarcinoma, to the “pure” rhabdoid carcinoma without an evident epithelial component [1,2]. The main differential diagnosis is with the malignant rhabdoid tumors (MRT), a neoplasm more common in childhood and characterized by genetic inactivation of *SMARCB1* (SNF5, INI-1), a component of the SWI/SNF chromatin remodelling complex or deletions of chromosome 22q [4-6]. The events involved in RCT pathogenesis, however, remain poorly elucidated [1-3]. In order to shed light on the molecular mechanisms underlying the stepwise rhabdoid carcinogenesis, we investigated the genetic and epigenetic alterations involved in two RCTs and compared with matched adenomas and normal mucosa.

## Materials and methods

Paraffin-embedded specimens of the neoplastic glandular and rhabdoid components of a pure and composite RCT were studied and compared to the matched normal mucosa and adenomas.

### Case I

A large and irregular carcinoma, measuring 10 × 10 cm and graded as T3N1M0, was diagnosed at the right colon and surgically removed in a 71-year-old woman at the Rummo Hospital, Benevento, Italy. Histologically, the tumor showed rhabdoid features without an apparent glandular component (pure RCT). Immunophenotipic, morphological and molecular findings supported its colorectal origin [2]. The patient was affected by essential hypertension and declared that her mother died of colorectal carcinoma (CRC). After surgery, she underwent adjuvant chemotherapy (Folfox for 3 months). Despite a target therapy as second line treatment (4 cycles of bevacizumab followed by 2 cycles of cetuximab), tumor dissemination to the peritoneum and liver occurred and the patient died only 8 months from surgery [2].

### Case II

**Figure 1:**
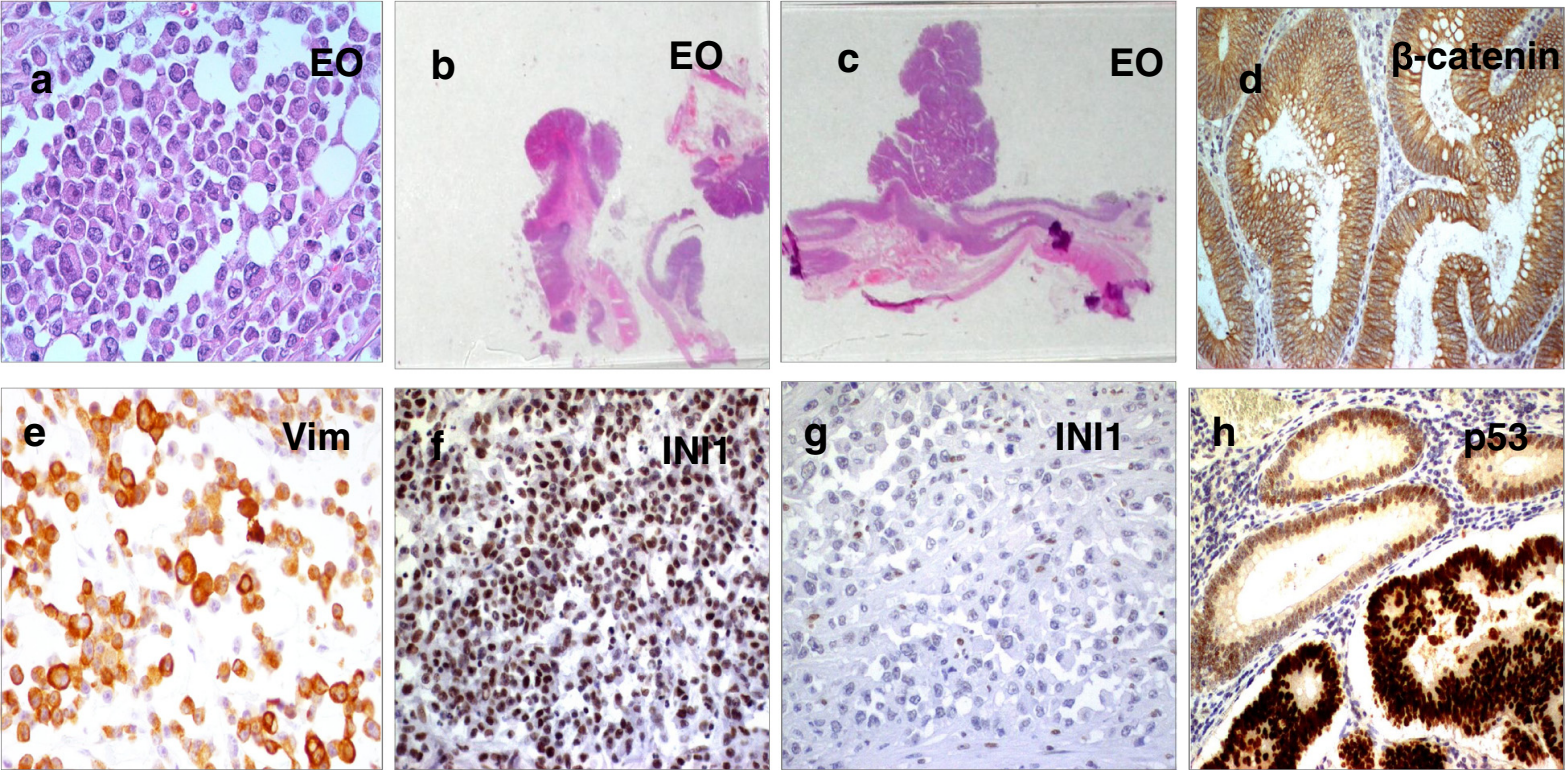
**Immunohistochemical markers of colon carcinoma with rhabdoid features, adjacent adenomas and normal mucosa.** (**a**) Hematoxylin&Eosin staining of the rhabdoid component in the composite RCT (case II) (**b**). Low-power view of tubular adenomas and (**c**) a larger dysplastic adenoma with a cancerized component contiguous to the main tumor mass of the composite RCT. (**d**) Membrane β-catenin staining in the large dysplastic adenoma adjacent to the composite RCT. (**e**) Intense and diffuse vimentin immunohistochemical staining in rhabdoid cells of pure RCT (case I) (**f**) Intense INI1 nuclear immunostaining in rhabdoid cells of case I (**g**) Loss of INI1 staining in the rhabdoid component of composite RCT showing appropriate staining of intratumoral lymphocytes serving as internal control. (**h**) A strong nuclear p53 staining marks the transition from adenoma to carcinoma in larger dysplastic polyps. Magnification (×200 or ×400).

The patient, a 73-year-old woman, was CRC diagnosed at the Legnago Hospital (Verona, Italy). The lesion, 10 × 8 cm in size, localized to the right colon, was graded as T4N1M0 [1]. Histologically, the tumor was heterogeneous, consisting of an adenocarcinoma associated with prominent rhabdoid features (composite RCT) (Figure 1a). Six tubular adenomas (TA) close to the carcinoma, were also present, among which the largest in size showed an infiltranting area of neoplastic cells (cancerized tubular adenoma, CTA) (Figure 1b, c). The area of rhabdoid dedifferentiation was approximately 40% of the entire tumor mass. The area of interest for each histological section was isolated and analyzed on the basis of its morphology. Patient’s anamnestic history revealed an essential hypertension and a meningioma at 31 years of age that was surgically removed; Only a sibling, among the proband’s first-degree relatives, was affected by CRC under 60 years of age; no family history nor other malignancies were reported. The patient underwent adjuvant chemotherapy (capecitabin and oxaliplatin) with no clinical benefits. She died for metastatic progression of the disease to the liver only 6 months after surgery [1].

### Immunohistochemical, methylation and DNA sequencing analysis

Four μm tick sections were used for routine stainings, immunohistochemistry and DNA extraction. Immunohistochemical analysis was performed as previously described [1,2] by using the following antibodies: VEGFR1 (sc-65442) and VEGFR2 (sc-101560); thymidylate synthase (TS) (sc-33679); APC (sc-896); (Santa Cruz Biotechnology, Santa Cruz, Ca, USA); HDACI (ab19845), HDACII (ab61216), HDACIII (ab-32117) (Abcam, Cambridge, UK); INI1 (25/BAF47) (DAKO Cytomation, Glostrup, Denmark). E-cadherin, 610405 and β-catenin (610153) (Transduction Laboratories, Lexington, KY, USA); cytokeratin 7 (CK7) clone-RN7; CK18-clone DC-10; CK19-clone b170; CK20-clone Ks 20.8; CK-Pan-clone AE1/AE3; epidermal growth factor receptor (EGFR)-clone EGFR.113; vimentin-clone VIM 3B4; desminclone DE-R-11; (Novocastra Laboratories, Newcastle, UK). p53 clone-Bp53-11; anti-MLH1 clone-M1; anti-MSH2 clone-G219-1129; (Ventana Medical Systems, Tucson, AZ, USA). Genomic DNA isolation and sodium bisulphite modification were carried out as reported [2]. The converted DNA was subjected to methylation specific PCR (MSP) using unmethylated and methylated controls in each reaction. The methylation levels (ratios of methylated to unmethylated and methylated DNA (M/U+M × 100) were determined from the relative band intensities and scored as follow: Negative (0-20% methylation); Low (>20-60%); High (>60%) [7]. Primers for promoter methylation analysis were designed and validated as reported [8]. A detailed description of the primer sets and MSP conditions has already been reported or will be provided upon request [2]. *EWS* rearrangement at the chromosome locus 22q12, *MYH*, *BRAF* and *KRAS* mutations were analyzed as previously reported [2,9].

## Results

### Immunohistochemical profile and predictive markers in RCTs

To evaluate whether common pathogenetic mechanisms underlie the development of RCT, we analyzed markers involved in colonic differentiation, epigenetic gene silencing, and predictors of drug resistance. Cytokeratin 20 (CK20), E-cadherin, CDX2 and β-catenin immunoreactivity were completely absent in the rhabdoid component and focally positive only in the glandular component (Figure 1d, and Table 1). Vimentin immunostaining was intense and diffuse in both cases, either in the rhabdoid and glandular neoplastic cells (Figure 1e, and Table 1). The matched normal mucosa and all adenomas tested, in contrast, were negative for vimentin and positive for epithelial markers (Table 1). Epigenetic and drug sensitivity markers, such as histone deacetylase isoforms (HDACs I-III) or vascular endothelial growth factor receptors 2 (VEGFR-2), were strongly or moderately positive in both cases, whereas thymidylate synthase (TS) and vascular endothelial growth factor receptors 1 (VEGFR-I) were focally immunoreactive (Table 1). INI1 immunostaining was weakly positive in normal mucosa, all adenomas and strongly positive in the pure RCT; conversely, it was focally positive in the glandular and totally negative in the rhabdoid component of the composite RCT (Figure 1f, g and Table 1).

**Table 1:** **Comparison of immunohistochemical markers between adjacent normal mucosa**, **tubular adenomas and RCT**

**Table 1**	**Pure RCT (case I)**		**Composite RCT (case II)**				
**Markers**	**NM1**	**RC**	**NM2**	**TA**	**CTA**	**GC**	**RC**
**CK20**	+++	neg.	+++	+++	+++	+	neg.
**CK7**	neg	neg.	neg.	neg.	neg.	neg.	neg.
**CDX2**	+++	neg.	+++	+++	+++	+	neg.
**E**-**cadherin**	++	neg.	++	++	++	+	neg.
**β-catenina **^**(a)**^	+	+	+	+	+	++	neg.
**APC (c-ter)**	++	++	++	++	+++	++	++
**p53**	neg.	++	neg.	+	+++	++	+
**Vimentin**	neg.	+++	neg.	neg	neg.	++	+++
**CK18**	+	+	+	++	++	+	++
**CK19**	++	++	++	++	++	+	++
**CK**-**Pan**	+++	++	+++	+++	+++	+++	+++
**HDAC1**	+	+++	+	+	++	++	+++
**HDAC2**	+	+++	+	+	++	++	+++
**HDAC3**	+	+++	+	+	++	++	+++
**VEGFR1**	neg.	neg.	neg.	+	+	neg.	neg.
**VEGFR2**	neg.	+++	neg.	neg.	neg.	++	++
**MLH1**	+++	neg.	+++	++	++	neg.	neg.
**MSH2**	+++	+	++	++	++	++	++
**TS**	+	+	+	++	++	+	+
**INI1**	+	+++	+	+	++	+	neg.

(+++): widespread occurrence, intense expression; (++): Varying distribution, moderate expression; (+): Limited occurrence, low expression; (−) no occurrence or negative. ^a^β-catenin nuclear localization; **Abbreviations**: Cytokeratin, CK; pure rhabdoid colorectal tumor, case I (RCTI); glandular (GC) and rhabdoid (RC) components of composite rhabdoid colorectal tumor, case II (RCTII); adjacent tubular adenomas (TA), cancerized and larger tubular adenoma (CTA) or normal mucosa (NM2) of case II. Case I, a pure RCT, is compared to the adjacent distant non-neoplastic mucosa (NM1); histone deacetylase, HDAC; vascular endothelial growth factor receptors, VEGFR; thymidylate synthase, TS.

### *BRAF* mutations and MSI are the predominant genetic alterations in RCT pathogenesis

**Figure 2:**
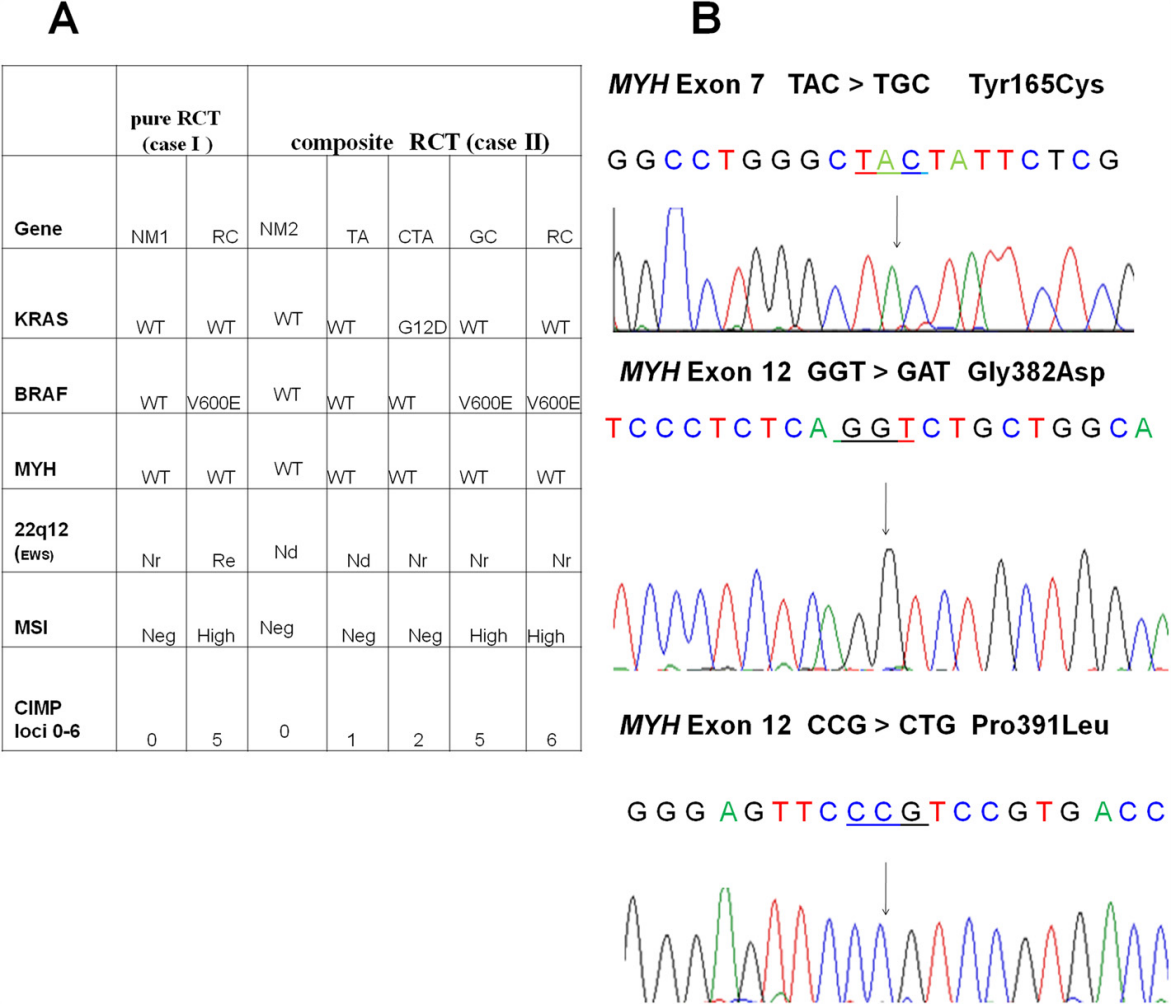
**Genetic and epigenetic survey of a composite and pure rhabdoid tumor.** (**A**). Genetic, cytogenetic and epigenetic analysis of the pure RCT (case I) and composite (case II) RTC. Tumor tissue of case II is characterized by a glandular (GC) and rhabdoid (RC) component whose molecular alterations are compared to the adjacent tubular adenomas (TA), the cancerized and larger tubular adenoma (CTA) or normal mucosa (NM2). Case I, a pure RCT, is compared to the adjacent distant non-neoplastic mucosa (NM1). Six CIMP loci *MLH1*, *CDKN2A*, *IGF2*, *SOX2*, *NEUROG1*, *RUNX3* are reported. (**B**) Representative sequencing analysis of *MYH* using DNA isolated from the rhabdoid tumor tissue shows no mutations at codons 165, 382 and 391, respectively. **Note**: Break-apart FISH assay was used to analyze 22q12 (*EWS*) rearrangement as reported in Ref 2. **Abbreviations**: Nd: Not determined; Nr: Normal or intact locus; Re: Rearranged locus; CIMP: CpG island methylator phenotype; MSI:microsatellite instability.

RCTs were characterized by the presence of the V600E *BRAF* mutation both in the rhabdoid and glandular component and by high microsatellite instability (MSI-H) due to *MLH1* inactivation [1,2]. (Figure 2A). To further investigate the genetic events involved in RCT development, we analyzed by FISH deletions or rearrangements at the *EWS* locus at 22q12 as they are detected in several MRTs [10]. The 22q12 locus was rearranged only in the pure RCT, but not in the composite tumor (Figure 2A). Subsequently, we compared the *BRAF*/*KRAS* mutational and the microsatellite stability status of the RCTs with the matched tubular adenomas (TAs) and normal mucosa. No *BRAF* mutations were found in all analyzed adenomas and normal adjacent mucosa (Figure 2A). The G12D *KRAS* mutation, in contrast, was found only in the larger dysplastic tubular adenoma with infiltrating carcinoma (CTA); only in this latter component an intense nuclear p53 expression was present, marking the transition from adenoma to carcinoma (Figure 1h, and Table 1). A negative immunostaining for MLH1 and a variable positivity for MSH2 were found in both RCTs (Table 1). MLH1 and MSH2 expression was positive in normal mucosa and in all adenomas tested, suggesting that high MSI was confined solely to RCTs (Figure 2A). The staining for APC (cytosolic), β-catenin (membrane) and CDX2 showed a normal pattern in all TAs tested and in normal mucosa (Table 1). These unexpected results prompted us to investigate whether other genetic alterations might be responsible for these lesions. To this goal we checked for *MYH* mutations at the most frequent hot spots, Y179C (exon7) and G396D and P391L (exon12) [11]. No mutations were found in RCTs and in normal mucosa (Figures 2A and 2B).

### Widespread CpG island promoter methylation is a feature of the rhabdoid trait

**Figure 3:**
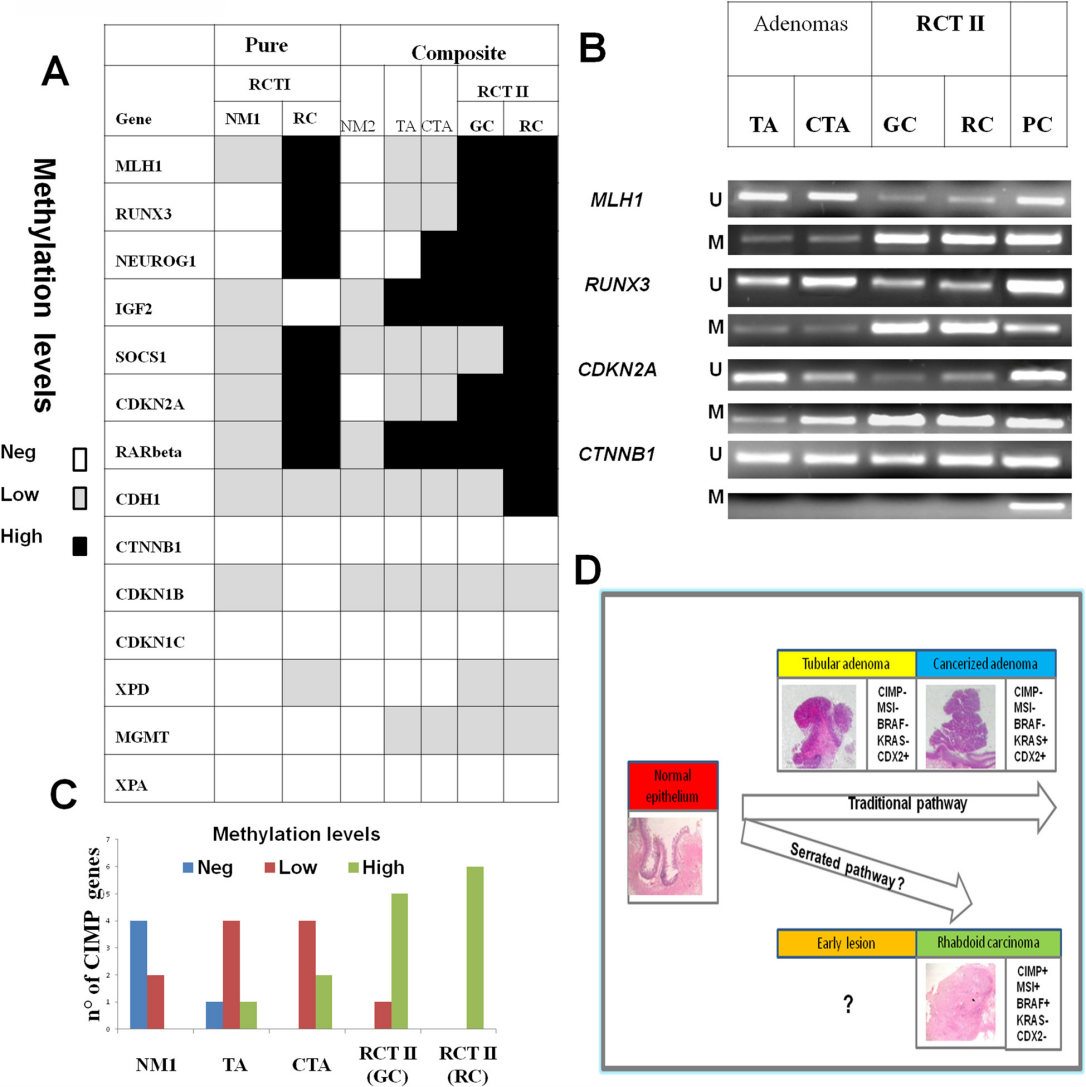
**Genome widespread CpG island promoter methylation is a molecular feature of RCT pathogenesis.** (**A**) The table reports the promoter methylation analysis carried out on 14 genes; six are the canonical, representative CIMP loci (*MLH1*, *CDKN2A*, *IGF2*, *SOX2*, *NEUROG1*, *RUNX3*); the others are either involved in cell cycle control as *CDKN1B* and *CDKN1C*; or in cell-cell adhesion as *CDH1* and *CTNNB1* or in DNA repair as *XPD*, *XPA*. The differential promoter methylation level in rhabdoid colorectal and adjacent lesions is shown. (**B**) Representative methylation specific PCR analysis at three CIMP loci (*MLH1*, *RUNX3*, *CDKN2A*) and *CTNNB1* (β-catenin) in case I; (PC) indicates positive unmethylated (U) or methylated (M) control, respectively. (**C**) Progressive accumulation of promoter methylation at six specific CIMP loci from normal mucosa, tubular adenomas and finally to composite RCT. (**D**) The schematic drawing illustrates the possible CRC pathogenetic mechanisms; rhabdoid CRC originates through an alternative pathway resembling the serrated pathway. **Abbreviations**: glandular (GC) and rhabdoid (RC) component of rhabdoid colorectal tumor case II (RCTII). NM: normal mucosa; TA: tubular adenoma; CTA: cancerized tubular adenoma.

The presence of a CpG island methylation profile in a tumor defines the CpG island methylator phenotype (CIMP), an epigenetic alteration considered as a novel genome instability event in CRC pathogenesis [12-14]. We assessed the DNA methylation levels of six CIMP markers, *MLH1*, *RUNX3*, *NEUROG1*, *IGF2*, *SOCS1* and *CDKN2A* in the normal mucosa, TA and RCT. Only in the RCTs, high levels of DNA methylation (from 80 to 100%) were found at the indicated loci (Figures 2A, 3A and 3B). Methylation at *SOCS1* and *CDH1* promoter regions was significantly higher in the rhabdoid than in the glandular component of the composite RCT (Figure 3A). CIMP loci methylation was only 30% in CTA, 16% in TA and relatively low or absent in the normal mucosa (Figure 3C). The methylation survey was extended to other genes not included in the CIMP panel, as their epigenetic changes in RCT pathogenesis have not been investigated so far. *RAR*β, *CTNNB1*, *CDKN1B*, *CDKN1C*, *XPD*, *XPA* and *MGMT*, among the others, did not show differential methylation levels during RCT progression; only *XPD* promoter methylation seemed to mark RCT specifically (Figure 3A).

## Discussion

Over the past twenty years, only seven cases of colorectal tumors with a rhabdoid phenotype have been reported [1-3]. These tumors are generally found in elderly patients at the proximal colon and show an aggressive behaviour characterized by an overall survival time shorter than 12 months [1,2]. The main differential diagnosis is with the malignant rhabdoid tumors (MRTs), a neoplasm more common in childhood characterized by genetic inactivation of *SMARCB1* (SNF5, INI-1), a component of the SWI/SNF chromatin remodelling complex or deletions at chromosome 22q [4-6]. Loss of epithelial markers, such as CK20, CDX2 and E-cadherin was observed in RCTs but neither in the matched normal mucosa nor adenomas. Although loss of CK20 and CDX2 is commonly observed in right-sided colorectal carcinoma [15,16] with high tumor grades and high microsatellite instability, the progressive increase of vimentin immunoreactivity may indicate that the sarcomatous dedifferentiation occurs in late stages of rhabdoid carcinogenesis and could be a crucial event in the transition from adenocarcinoma to RCT [1-3]. Usually, RCTs express high levels of EGFR [1,2], a finding that is recurrent also in **other subgroups** of highly aggressive **CRCs**. These results suggest that a combined assessment of CDX2, Vimentin and EGFR may be of clinical value to make a differential diagnosis, to predict a poor patients’ outcome or to choose the best fit biological therapy [17,18]. INI1 loss-of-function mutations have been identified in pediatric MRTs, whereas their role in adult extra-renal rhabdoid tumors is still elusive [5,6]. Although loss of INI1 expression is a constant finding in MRT of soft tissues, kidney or central nervous system (CNS), INI1 staining was positive in the normal mucosa, adenomas and intensely in pure RCT, and negative in the composite RCT. Loss of INI1 in MRT is mainly due to mutations and/or deletions of the 22q11.2 locus; alternatively, it may be due to epigenetic events. The 22q12 locus was normal in composite RCT with lack of INI1 whereas it was rearranged in pure RCT with an intense INI1 staining, supporting the hypothesis of gene inactivation possibly by epigenetic mechanisms. The EWS gene is located on chromosome 22q12 and its translocation with members of the ETS families is a recurrent alteration in Ewing sarcoma, although other EWS rearrangements or deletions have been identified in different pathologies including composite rhabdoid tumors of the endometrium [10,19]. A rearrangement was detected in the pure RCT, the epithelial origin of which has already been reported [2]. Although chromosome 22 alterations are common in MRTs, their role in CRC and RCT is still unknown and further studies are required [19].

The APC/β-catenin pathway seems to be not affected ruling out that RTC originates through the traditional adenoma–carcinoma pathogenetic pathway underlying most CRCs (Figure 3D). A *KRAS* mutation was found only in the CTA, while nuclear p53 accumulation was observed exclusively in its cancerized component. Interestingly, no mutations were found in the base excision repair gene *MYH*, whose mutations predispose to an hereditary colorectal cancer syndrome defined “*MYH* associated polyposis” (MAP) characterized by multiple adenomas mainly at the right colon [9,11]. While our data do not exclude that RCT may bear causative mutations in other yet unknown loci, they, however, confirm that *MYH* is not responsible either for the precursor or tumor lesions.

Microsatellite instability (MSI) due to deficiency of the mismatch repair system has been described in about 15-20% of sporadic CRCs, characterized by poor differentiation, infiltrating lymphocytes, a mucinous phenotype and a more proximal localization than inherited HNPCCs [12-14]. *MLH1* epigenetic silencing is the most frequent event responsible for MSI and is associated with *BRAF* mutations in the serrated pathway [13,20]. In our RTCs, MLH1 staining was negative, while it was nuclear and diffuse in all adenomas and adjacent normal mucosa. Remarkably, this expression profile was associated with the presence of *BRAF* mutations in the same tissues, strongly supporting our hypothesis that RTC does not arise through the traditional adenoma-carcinoma sequence (Figure 3D). Rather, it suggests that RCT may evolve through the serrated pathway [14]. At the best of our knowledge, these genetic alterations have not been reported in MRT suggesting a colonic origin of RCT.

*MLH1* silencing is usually associated with hypermethylation at the CpG islands in multiple gene promoters [21]. Interestingly, we found a progressive accumulation of promoter methylation at specific CIMP loci and additional genes from the normal mucosa to tubular adenoma, CTA and RCT. A CpG island methylation threshold seems to be required for repression of *MLH1* and other important CIMP loci in the composite RCT: a promoter methylation above 60% was, in fact, required for *MLH1* down-regulation, as reported [21,22]. At the right colon, a CIMP+ phenotype and microsatellite instability (CIMP+/MSI+) may predispose to RCT development, in agreement with recent studies reporting that CIMP+/MSI+ subtypes have a worse clinical behaviour and prognosis than patients with CIMP-/MSI+ [14].

## Conclusions

Clinical and molecular features suggest that RCT may be considered a distinct colonic entity and could benefit from specific treatments with novel biological molecules. Right-sided RCT could be characterized by epigenetic events similar to those occurring in the serrated pathway with a marked epithelial-mesenchymal transition. Further investigations involving more cases, albeit rare, are mandatory to support this hypothesis.

## Consent

"Written informed consent was obtained from the patient for publication of this Case Report and any accompanying images. A copy of the written consent is available for review by the Editor-in-Chief of this journal."

## Competing interests

The authors declare that they have no competing interests.

## Authors’ contributions

MP, AR conceived and designed the study. MP, LS, CZ, CV, AF, FF, EM performed the experiments. MP, LS, CZ, ADB, GL, BD, EM, NN, RV and VC analyzed and interpreted the data. MP, AR and VC wrote the paper. All authors reviewed and approved the final manuscript.

## References

[B1] Remo A Zanella C Molinari E Talamini A Tollini F Piacentini P Battaglia P Baritono E Bonetti A Lanza F Fasolin A Manfrin E Vendraminelli R Rhabdoid carcinoma of the colon, a distinct entity with a very aggressive behaviour. A case report, associated with a polyposis coli and review of the literature Int J Surg Pathol 2012 20 185 190 21791485 10.1177/1066896911415405

[B2] Pancione M Di Blasi A Sabatino L Fucci A Dalena AM Palombi N Carotenuto P Aquino G Daniele B Normanno N Colantuoni V A novel case of rhabdoid colon carcinoma associated with a positive CpG island methylator phenotype and BRAF mutation Hum Pathol 2011 42 1047 1052 10.1016/j.humpath.2010.10.016 21315413

[B3] Tóth L Nemes Z Gomba S Asztalos L Molnár C András C Szentirmay Z Molnár P Primary rhabdoid cancer of the ileum: a case report and review of the literature Pathol Res Pract 2010 206 110 115 10.1016/j.prp.2009.02.013 19369011

[B4] Fletcher C Unni K Mertens F WHO Pathology and Genetics of Tumours of Soft Tissue and Bone 2002 France: Lyon IARC Press

[B5] Roberts CW Biegel JA The role of SMARCB1/INI1 in development of rhabdoid tumor Cancer Biol Ther 2009 8 412 416 10.4161/cbt.8.5.8019 19305156 PMC2709499

[B6] Biegel JA Zhou JY Rorke LB Stenstrom C Wainwright LM Fogelgren B Germ-line and acquired mutations of INI1 in atypical teratoid and rhabdoid tumors Cancer Res 1999 59 74 79 9892189

[B7] Breault JE Shiina H Igawa M Ribeiro-Filho LA Deguchi M Enokida H Urakami S Terashima M Nakagawa M Kane CJ Carroll PR Dahiya R Methylation of the g-Catenin Gene Is Associated With Poor Prognosis of Renal Cell Carcinoma Clin Cancer Res 2005 11 557 564 15701841

[B8] Pancione M Sabatino L Fucci A Carafa V Nebbioso A Forte N Febbraro A Parente D Ambrosino C Normanno N Altucci L Colantuoni V Epigenetic silencing of Peroxisome Proliferator-Activated Receptor γ is a biomarker for colorectal cancer progression and adverse patients' outcome PLoS One 2010 5 e14229 10.1371/journal.pone.0014229 21151932 PMC2997072

[B9] van Puijenbroek M Nielsen M Tops CM Halfwerk H Vasen HF Weiss MM van Wezel T Hes FJ Morreau H Identification of patients with (atypical) MUTYH-associated polyposis by *KRAS*2 c.34G > T prescreening followed by MUTYH hotspot analysis in formalin-fixed paraffin-embedded tissue Clin Cancer Res 2008 14 139 142 10.1158/1078-0432.CCR-07-1705 18172263

[B10] Donner LR Wainwright LM Zhang F Biegel JA Mutation of the INI1 gene in composite rhabdoid tumor of the endometrium Hum Pathol 2007 38 935 939 10.1016/j.humpath.2006.12.003 17376508 PMC1963314

[B11] Lubbe SJ Di Bernardo MC Chandler IP Houlston RS Clinical implications of the colorectal cancer risk associated with MUTYH mutation J Clin Oncol 2009 24 3975 3980 19620482 10.1200/JCO.2008.21.6853

[B12] Issa JP Colon Cancer: It’s CIN or CIMP Clin Cancer Res 2008 14 5939 5940 10.1158/1078-0432.CCR-08-1596 18829469

[B13] Leggett B Whitehall V Role of the serrated pathway in colorectal cancer pathogenesis Gastroenterology 2010 138 2088 2100 10.1053/j.gastro.2009.12.066 20420948

[B14] Pancione M Remo A Colantuoni V Genetic and epigenetic events generate multiple pathways in colorectal cancer progression Patholog Res Int 2012 2012 509348 22888469 10.1155/2012/509348 PMC3409552

[B15] McGregor DK Wu TT Rashid A Luthra R Hamilton SR Reduced expression of cytokeratin 20 in colorectal carcinomas with high levels of microsatellite instability Am J Surg Pathol 2004 28 712 718 10.1097/01.pas.0000126757.58474.12 15166663

[B16] Baba Y Nosho K Shima K Freed E Irahara N Philips J Meyerhardt JA Hornick JL Shivdasani RA Fuchs CS Ogino S Relationship of *CDX2* loss with molecular features and prognosis in colorectal cancer Clin Cancer Res 2009 15 4665 4673 10.1158/1078-0432.CCR-09-0401 19584150 PMC2777758

[B17] El Demellawy D Khalifa MA Ismiil N Wong S Ghorab Z Primary colorectal small cell carcinoma: a clinicopathological and immunohistochemical study of 10 cases Diagn Pathol 2007 2 35 10.1186/1746-1596-2-35 17803816 PMC2034542

[B18] Dekanić A Dintinjan RD Budisavljević I Pećanić S Butorac MŽ Jonjić N Strong nuclear EGFR expression in colorectal carcinomas is associated with cyclin-D1 but not with gene EGFR amplification Diagn Pathol 2011 6 108 10.1186/1746-1596-6-108 22050898 PMC3225321

[B19] Hollmann TJ Hornick JL INI1-deficient tumors: diagnostic features and molecular genetics Am J Surg Pathol 2011 35 47 63 10.1097/PAS.0b013e31822b325b 21934399

[B20] Deng G Bell I Crawley S Gum J Terdiman JP Allen BA Truta B Sleisenger MH Kim YS *BRAF* mutation is frequently present in sporadic colorectal cancer with methylated hMLH1, but not in hereditary nonpolyposis colorectal cancer Clin Cancer Res 2004 10 10191 10195 10.1158/1078-0432.ccr-1118-3 14734469

[B21] Wong JJ Hawkins NJ Ward RL Hitchins MP Methylation of the 3p22 region encompassing MLH1 is representative of the CpG island methylator phenotype in colorectal cancer Mod Pathol 2011 24 396 411 10.1038/modpathol.2010.212 21102416

[B22] Furukawa T Konishi F Masubuchi S Shitoh K Nagai H Tsukamoto T Densely Methylated *MLH1* Promoter Correlates With Decreased mRNA Expression in Sporadic Colorectal Cancers Genes Chromosomes Cancer 2002 35 1 10 10.1002/gcc.10100 12203784

